# Analytical Solution of Time-Periodic Electroosmotic Flow through Cylindrical Microchannel with Non-Uniform Surface Potential

**DOI:** 10.3390/mi10080498

**Published:** 2019-07-26

**Authors:** Aminul Islam Khan, Prashanta Dutta

**Affiliations:** School of Mechanical and Materials Engineering, Washington State University, Pullman, WA 99164-2920, USA

**Keywords:** time-periodic electroosmotic flow, heterogeneous surface charge, cylindrical microchannel, stream function, micro-mixing

## Abstract

Time-periodic electroosmotic flow (EOF) with heterogeneous surface charges on channel walls can potentially be used to mix species or reagent molecules in microfluidic devices. Although significant research efforts have been placed to understand different aspects of EOF, its role in the mixing process is still poorly understood, especially for non-homogeneous surface charge cases. In this work, dynamic aspects of EOF in a cylindrical capillary are analyzed for heterogeneous surface charges. Closed form analytical solutions for time-periodic EOF are obtained by solving the Navier–Stokes equation. An analytical expression of induced pressure is also obtained from the velocity field solution. The results show that several vortices can be formed inside the microchannel with sinusoidal surface charge distribution. These vortices change their pattern and direction as the electric field change its strength and direction with time. In addition, the structure and strength of the vorticity depend on the frequency of the external electric field and the size of the channel. As the electric field frequency or channel diameter increases, vortices are shifted towards the channel surface and the perturbed flow region becomes smaller, which is not desired for effective mixing. Moreover, the number of vorticities depends on the periodicity of the surface charge.

## 1. Introduction

Lab-on-a-chip microfluidic devices are utilized for numerous applications such as DNA sequencing, synthesis, crystallization, polymerization, and drug discovery. These applications require a number of unit operations such as pumping [1], mixing [2], etc. Among various functionalities, mixing in microfluidic devices is very challenging due to (ultra-low Reynolds number) creeping flow [3]. The quick mixing of species is key to reducing analysis time in a microfluidic device for its widespread application in the biomedical field [4]. To date, several mixing strategies have been proposed for microfluidic systems with different degrees of successes (see reviews [5,6,7]). Based on the methods used to achieve mixing, micromixers are generally classified as being passive or active. In passive micromixers, the mixing process depends entirely on diffusion; meanwhile, in active micromixers, the mixing process is accelerated by an external disturbance [6,8]. Although the passive mixing offers some benefits such as no external power requirements, it is not very effective in microfluidic devices because of the diffusion-based very slow mixing process. Active mixing has the potential to eliminate the inherent drawbacks of slow mixing using an acoustic, magnetic, or electrokinetic forcing [9]. Among various active micromixers (see reviews [2,5]), electrokinetic-based micromixers are preferred owing to their numerous advantages including ease of fabrication, no moving parts, ease of control, high reliability and repeatability, and quiet operation.

Electroosmotic mixing can be enhanced with an alternating electric field. Alternating electric field-based time-periodic electroosmotic flow (EOF) has been extensively studied with uniform surface charges for various types of channels such as parallel plate [10], circular [11], rectangular [12], and annulus [13] channels, etc. Oddy et al. [14] found that the rapid stretching and folding of fluid interfaces, induced by an alternating electric field, are able to stir fluid streams at a very low Reynolds number (Re < 1). Glasgow et al. [15] found that better mixing can be achieved in a T-junction channel with two out-of-phase alternating electric fields. The out-of-phase alternating electric field induces the oscillation of the fluid interface at the junction of the two inlet channels, which increases the contact surface area between the two fluid streams for significantly better mixing efficiency [16]. However, the alternating electric field-based EOF fails to take advantage of the vortex to enhance the mixing due to the unidirectional flow at any particular time.

The use of a nonhomogeneous channel surface is another way to improve electroosmotic mixing performance. The effects of a heterogeneous surface charge have also been extensively studied in direct current (DC) EOF. For example, Ajdari [17] theoretically showed that a surface with a sinusoidal charge can create vorticities within the bulk flow. Vortices formation within the bulk flow was also shown by Horiuchi et al. [18] for a step change in the surface charge in a DC EOF. Through a numerical investigation, Erickson and Li [19] showed that surface heterogeneity increases the mixing efficiency and reduces the required mixing length. Qian and Bau [20] presented a theoretical study of electroosmotic flows driven by a uniform electric field in a two-dimensional conduit with non-uniform surface potential distribution.

In microfluidic devices, surface heterogeneity can be introduced intentionally through micromanufacturing technology, such as microcontact printing or rapid prototyping. For instance, Biddiss et al. [4] manufactured several micromixers with heterogenous surface charge configurations by rapid prototyping for the quick mixing of species. Their analysis showed that heterogeneously charged micromixers significantly improve the mixing efficiency as compared to homogeneously charged micromixers. Strook et al. [21] also used microcontact printing to vary the surface charge in directions parallel and perpendicular to the applied electric field. They found that surface charge variation in the parallel direction generates recirculating flow, while surface charge variation in the perpendicular direction creates multidirectional flow. Electrokinetic characteristics of a microchannel surface can also be modulated through the dynamic or static coating of proteins, DNA, colloids, and/or nanosized particles on the surface. For example, Norde and Rouwendal [22] studied the change of surface charge by the protein adsorption on a glass surface. Wei et al. [23] generated non-uniform zeta potential distribution by periodically attaching DNA molecules in the microfluidic channel surface for increased DNA–DNA hybridization. The aforementioned studies showed that DC EOF with a heterogeneous surface charge can increase the mixing efficiency while reducing the mixing length by creating vorticity. However, a DC EOF fails to take advantages offered by an alternating electric field.

Thus, the primary focus of this work is to further increase the efficiency of electroosmotic mixing by introducing an alternating electric field in a heterogeneously charged channel. Through a numerical investigation, Lee et al. [24] demonstrated various kinds of flow circulation in a circular slit with time-periodic EOF for non-uniform zeta potential distribution along the microchannel walls. Luo [25] numerically investigated the transient electroosmotic flow induced by an alternating electric field with patch-wise surface heterogeneities in a planner microchannel. Tang et al. [26] studied the pulsating electroosmotic flow in a planar microchannel with non-uniform surface charges by the lattice Boltzmann method. These simulation results showed that time-periodic EOF with a heterogeneous surface can combine the advantages of the alternating electric field and the heterogeneous surface charge. However, no generalized analytical model exists for time-periodic EOF with non-uniform zeta potential in cylindrical systems. An analytical solution could help to find the optimum micromixer design. Motivated by the growing interest in time-periodic EOF as a reliable non-mechanical strategy to mix species in microfluidic devices, in this paper, a theoretical approach is presented. Specifically, we studied a time-periodic EOF field in a cylindrical microchannel with heterogeneous surface charge distribution. A closed-form analytical solution was obtained for the time-periodic electroosmotic flow velocity by solving the Navier–Stokes equations. In addition, an expression for pressure distribution was also obtained for time-periodic electroosmotic flow.

## 2. Mathematical Model

### 2.1. Governing Equations for Time-Periodic EOF in a Cylindrical Microchannel

**Figure 1:**
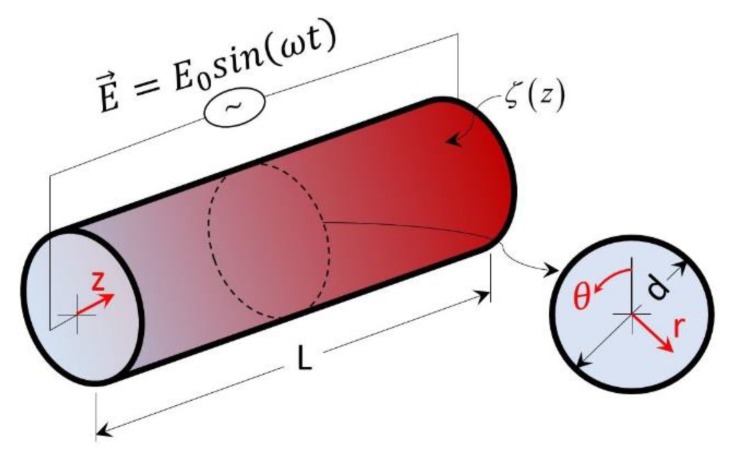
Schematic of a cylindrical microchannel with an associated polar coordinate system.

Figure 1 shows a schematic for time-periodic EOF in a cylindrical microchannel, where an alternating electric field is applied along the channel length. For generality, the model was developed such that the surface zeta potential can take any periodic distribution (e.g., sinusoidal, square) along the length of the channel, but there is no variation in zeta potential along the circumferential direction.

The application of a time-periodic external electric field results in a net body force on the free ions within the electric double layer (EDL), inducing a time-periodic bulk fluid motion. The motion of the fluid inside the channel can be governed by the incompressible Navier–Stokes equation [10]:

(1) $$\rho \left[\frac{\partial \vec{V}}{\partial t}+\left(\vec{V}\cdot \nabla \right)\vec{V}\right]=-\nabla P+\mu \nabla^{2}\vec{V}+\rho_{e}\vec{E}$$

where ρ and μ are the density and viscosity of the fluid, respectively; P is the pressure; $\rho_{e}$ is the charge density; and $\vec{E}$ is the applied electric field. The applied electric field can be expressed as $\vec{E}=E_{z}\hat{z}=E_{0}\sin\left(\omega t\right)\hat{z}$, where $\hat{z}$ is the unit vector in the axial (*z*) direction and $E_{0}$ is the reference electric field. In addition, the mass conservation equation can be expressed as:

(2) $$\frac{\partial \rho }{\partial t}+\nabla \cdot \left(\rho \vec{V}\right)=0.$$

In general, for an incompressible fluid, density is assumed to be a constant. Thus, for the axisymmetric flow scenario ($v_{\theta }=0$) presented here, the mass conservation equation can be reduced to:

(3) $$\frac{1}{r}\frac{\partial }{\partial r}(rv_{r})+\frac{\partial v_{z}}{\partial z}=0.$$

where $v_{r}$ and $v_{z}$ are the velocity components in the radial and axial direction, respectively. For the axisymmetric case, the radial and axial velocity can be related to the stream function, ψ as follows:

(4) $$v_{r}=\frac{1}{r}\frac{\partial \psi }{\partial z},v_{z}=-\frac{1}{r}\frac{\partial \psi }{\partial r}.$$

### 2.2. Analysis of Time-Periodic EOF in a Cylindrical Microchannel

Our next objective is to simplify the Navier–Stokes equations. In most microfluidic applications, the buffer solutions have a concentration of the order of mM, which results in a very thin EDL. The bulk fluid flow outside of the EDL region can be modeled by dropping the electroosmotic body force, $\rho_{e}\vec{E}$, and by introducing a Helmholtz–Smoluchowski slip boundary condition at the channel wall [24]:

(5) $${\vec{V}}_{r=r_{0}}=-\frac{\varepsilon \zeta \left(z\right)}{\mu }\vec{E}$$

where ε is the permittivity and ζ(z) is the surface potential distribution along the length of the conduit. This slip boundary condition was originally developed for steady-state EOF, but it can still be used for time-periodic EOF because the applied electric field frequency ($10^{2}∼10^{5}\;\mathrm{Hz}$) is less than the charge relaxation frequency ($10^{6}∼10^{8}\;Hz$) [27]. Moreover, the Helmholtz–Smoluchowski formulation-based slip boundary condition is widely used for nonhomogeneous surface charges as well as time-periodic EOF analysis [3,24,28,29,30]. Thus by dropping the body force, the component form of equations of motion are:

(6a) $$\rho \left(\frac{\partial v_{r}}{\partial t}+v_{r}\frac{\partial v_{r}}{\partial r}+v_{z}\frac{\partial v_{r}}{\partial z}\right)=-\frac{\partial P}{\partial r}+\mu \left[\frac{\partial }{\partial r}\left\{\frac{1}{r}\frac{\partial }{\partial r}\left(rv_{r}\right)\right\}+\frac{\partial^{2}v_{r}}{\partial z^{2}}\right],$$

(6b) $$\rho \left(\frac{\partial v_{z}}{\partial t}+v_{r}\frac{\partial v_{z}}{\partial r}+v_{z}\frac{\partial v_{z}}{\partial z}\right)=-\frac{\partial P}{\partial z}+\mu \left[\frac{1}{r}\frac{\partial }{\partial r}\left(r\frac{\partial v_{z}}{\partial r}\right)+\frac{\partial^{2}v_{z}}{\partial z^{2}}\right].$$

Here, the θ-component of the momentum equation is discarded due to axisymmetric flow, as there is no driving force in the θ-direction. In general, the advection effects are negligible in EOF due to the low Reynolds number [31,32]. For example, if water (ρ=1000 $\mathrm{kg}\cdot m^{-3}$ and μ=0.001 $\mathrm{kg}\cdot m^{-1}\cdot s^{-1}$) flows through a 100 μm diameter cylindrical microchannel with an axial velocity of 1 $\mathrm{mm}\cdot s^{-1},$ the resulting Reynolds number would be Re=0.1. Thus, by dropping the advective term, eliminating P between Equation (6), and introducing the stream function ψ, we get:

(7) $$\left(D-\frac{1}{\vartheta }\frac{\partial }{\partial t}\right)D\psi =0$$

where ϑ is the kinetic viscosity and D denotes the following operator:

(8) $$D\equiv \frac{\partial^{2}}{\partial r^{2}}-\frac{1}{r}\frac{\partial }{\partial r}+\frac{\partial^{2}}{\partial z^{2}}.$$

The commutative properties of operators D and $D-\frac{1}{\vartheta }\frac{\partial }{\partial t}$ lead us to separate the stream function, ψ, into two parts [32,33] as $\psi =\psi_{1}+\psi_{2}$, where $\psi_{1}$ satisfies the equation

(9) $$D\psi_{1}=0$$

and $\psi_{2}$ satisfies

(10) $$\left(D-\frac{1}{\vartheta }\frac{\partial }{\partial t}\right)\psi_{2}=0.$$

Considering periodic boundary conditions in the upstream (z=0) and downstream (z=L) regions, we assume wave-like solutions of elliptic Equations (9) and (10), which can be given as:

(11a) $$\psi_{1}=\sum_{n=-\infty }^{\infty }\varphi_{1,n}\left(r\right)e^{ik_{n}z}e^{i\omega t},$$

(11b) $$\psi_{2}=\sum_{n=-\infty }^{\infty }\varphi_{2,n}\left(r\right)e^{ik_{n}z}e^{i\omega t}.$$

Here, $\phi_{1,n}$ and $\phi_{2,n}$ are eigenfunctions, ω is the temporal angular frequency, and $k_{n}$ is the eigenvalue for the spatially periodic process. Thus, Equation (11) provides a general solution for axially periodic boundary conditions [32,33]. The values of $k_{n}$ can be given as 2πn/L, where n is an integer and L is the length of the microfluidic conduit. The eigenfunctions $\phi_{1,n}$ and $\phi_{2,n}$ are solutions of the following differential equations:

(12a) $$\frac{d^{2}\varphi_{1,n}}{dr^{2}}-\frac{1}{r}\frac{d\varphi_{1,n}}{dr}-k_{n}^{2}\varphi_{1,n}=0,$$

(12b) $$\frac{d^{2}\phi_{2,n}}{dr^{2}}-\frac{1}{r}\frac{d\phi_{2,n}}{dr}-l_{n}^{2}\phi_{2,n}=0,$$

satisfying the boundary conditions at the tube wall. In Equation (12b), $l_{n}^{2}=k_{n}^{2}+i\omega /\vartheta$. The general solutions of Equation (12a,b) can be obtained as

(13a) $$\phi_{1,n}=r\left[A_{1,n}I_{1}\left(k_{n}r\right)+B_{1,n}K_{1}\left(k_{n}r\right)\right],$$

(13b) $$\phi_{2,n}=r\left[A_{2,n}I_{1}\left(l_{n}r\right)+B_{2,n}K_{1}\left(l_{n}r\right)\right],$$

respectively, where $I_{1}\left(r\right)$ and $K_{1}\left(r\right)$ are modified Bessel functions of the first and second kind of order 1; $A_{1,n}$, $A_{2,n}$, $B_{1,n}$, $B_{2,n}$ are coefficients. Thus, the stream function, ψ, can be given as follows:

(14) $$\psi =\sum_{n=-\infty }^{\infty }r\left[A_{1,n}I_{1}\left(k_{n}r\right)+B_{1,n}K_{1}\left(k_{n}r\right)+A_{2,n}I_{1}\left(l_{n}r\right)+B_{2,n}K_{1}\left(l_{n}r\right)\right]\;e^{ik_{n}z}e^{i\omega t}.$$

From the stream function solution (Equation (14)), the radial and axial velocity components can be obtained using Equation (4):

(15a) $$v_{r}=\sum_{n=-\infty }^{\infty }\left[A_{1,n}I_{1}\left(k_{n}r\right)+B_{1,n}K_{1}\left(k_{n}r\right)+A_{2,n}I_{1}\left(l_{n}r\right)+B_{2,n}K_{1}\left(l_{n}r\right)\right]\;ik_{n}e^{ik_{n}z}e^{i\omega t},$$

(15b) $$v_{z}=\sum_{n=-\infty }^{\infty }\left[-k_{n}A_{1,n}I_{0}\left(k_{n}r\right)+k_{n}B_{1,n}K_{0}\left(k_{n}r\right)-l_{n}A_{2,n}I_{0}\left(l_{n}r\right)+l_{n}B_{2,n}K_{0}\left(l_{n}r\right)\right]\;e^{ik_{n}z}e^{i\omega t}.$$

Our next objective is to find coefficients for velocity distribution. The velocity components $v_{r}$ and $v_{z}$ must be finite everywhere. This condition requires that the velocity components do not contain the function $K_{1}\left(k_{n}r\right)$; hence, $B_{1,n}=B_{2,n}=0$. At the channel wall ($r=r_{0}=d/2$), the boundary conditions for radial and axial velocity become no penetration to the wall ($v_{r}=0$) and Helmholtz–Smoluchowski slip velocity ($v_{z}=-\varepsilon \zeta \left(z\right)E_{0}e^{i\omega t}/\mu$), respectively. The first boundary condition requires that $A_{2,n}=-A_{1,n}I_{1}\left(k_{n}r_{0}\right)/I_{1}\left(l_{n}r_{0}\right)$. Using the second Helmholtz–Smoluchowski boundary condition and applying complex Fourier analysis, one can find the remaining coefficient using the equation $A_{1,n}=-\frac{\varepsilon E_{0}I_{1}\left(l_{n}r_{0}\right)}{\mu \left[-k_{n}I_{0}\left(k_{n}r_{0}\right)I_{1}\left(l_{n}r_{0}\right)+l_{n}I_{1}\left(k_{n}r_{0}\right)I_{0}\left(l_{n}r_{0}\right)\right]}\;\frac{1}{L}\int_{0}^{L}\zeta \left(z\right)e^{-ik_{n}z}dz$. Thus, the final forms of radial and axial velocity can be given as

(16a) $$v_{r}=\sum_{n=-\infty }^{\infty }A_{1,n}\left[I_{1}\left(k_{n}r\right)-\frac{I_{1}\left(k_{n}r_{0}\right)}{I_{1}\left(l_{n}r_{0}\right)}I_{1}\left(l_{n}r\right)\right]\;ik_{n}e^{ik_{n}z}e^{i\omega t},$$

(16b) $$v_{z}=\sum_{n=-\infty }^{\infty }A_{1,n}\left[-k_{n}I_{0}\left(k_{n}r\right)+l_{n}\frac{I_{1}\left(k_{n}r_{0}\right)}{I_{1}\left(l_{n}r_{0}\right)}I_{0}\left(l_{n}r\right)\right]\;e^{ik_{n}z}e^{i\omega t},$$

respectively. The imaginary part of these equations provides flow velocity corresponds to the external electric field, $\vec{E}=E_{0}sin\left(\omega t\right)=Im\left(E_{0}e^{i\omega t}\right)$, which we considered throughout the paper. Even though Equation (16) provides the analytical solution for time-periodic electroosmotic flow in a heterogeneously charged circular shaped channel, it cannot be used for time-periodic electroosmotic flow in a homogeneously charged channel since we started our analysis for a general solution (Equation (11)) with periodicity in the axial (*z*) direction. Thus, to obtain time-periodic electroosmotic velocity in a homogenously charged microchannel, one has to drop the z-dependency in the general solution and governing equations (Equation (6)) and follow the method described in this work.

## 3. Results and Discussion

In this section, results are provided for a sinusoidal surface charge distribution, although the general solution presented in the aforementioned section can be applied for any periodic surface charge distribution along the tube. The sinusoidal surface charge distribution can be given as:

(17) $$\zeta \left(z\right)=\zeta_{0}\sin\left(qz\right)$$

where $\zeta_{0}$ is the amplitude (reference) of the surface charge and q is the angular frequency of the surface charge distribution. With this charge distribution, the coefficient for Equation (16) can be found as follows:

(18) $$A_{1,n}=-\frac{\varepsilon E_{0}I_{1}\left(l_{n}r_{0}\right)}{\mu \left[-k_{n}I_{0}\left(k_{n}r_{0}\right)I_{1}\left(l_{n}r_{0}\right)+l_{n}I_{1}\left(k_{n}r_{0}\right)I_{0}\left(l_{n}r_{0}\right)\right]}\;\frac{\zeta_{0}}{L}\left[\frac{\left\{ik_{n}\sin\left(qL\right)+q\cos\left(qL\right)\right\}e^{-ik_{n}L}-q}{k_{n}^{2}-q^{2}}\right]$$

**Figure 2:**
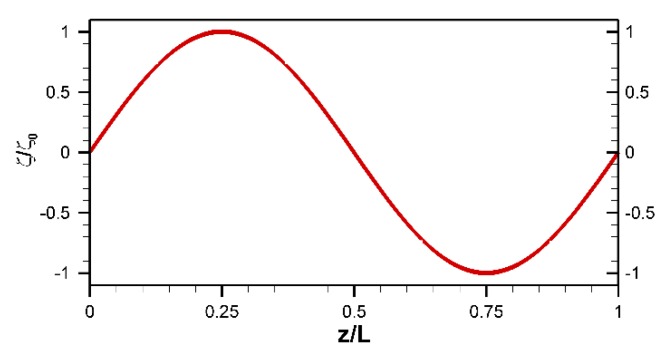
Schematic of the zeta potential distribution with a single period.

Throughout this section, unless otherwise stated, the value of q is considered as 2π/L, which corresponds to a single period in the surface charge distribution as shown in Figure 2.

### 3.1. Velocity Profiles at Various Nondimensional Times

**Figure 3:**
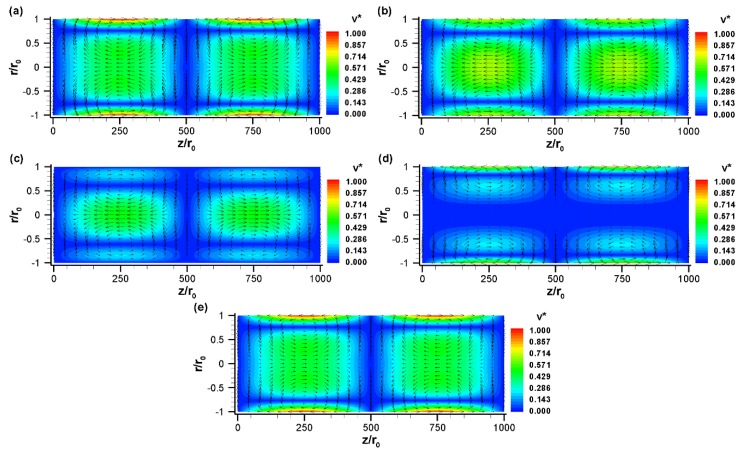
The normalized velocity,${\vec{v}}^{*}=\vec{V}/u_{HS}$ (vector plot), and its magnitude, $v*=\left|\vec{V}\right|/u_{HS}$ (contour plot), with sinusoidal zeta potential distribution at various nondimensional times: (**a**) ωt=π/2, (**b**) ωt=3π/4, (**c**) ωt=π, (**d**) ωt=5π/4, and (**e**) ωt=3π/2. The vector plot shows the direction of fluid motion whereas the contour plot shows the magnitude of the velocity. Here, $u_{HS}=-\varepsilon \zeta E_{0}/\mu$, $E_{0}=10$ kV/m, $\zeta_{0}=-100$ mV, $r_{0}=50$ μm, L=5 cm, q=2π/L, f=1.6 kHz (Ω=25). Fluid properties are taken for water at 20 °C.

Figure 3 illustrates the velocity vector at different nondimensional times for a sinusoidal zeta potential distribution along the channel surface with a reference potential of −100 mV. When the zeta potential is changed along the channel wall, the driving force in electroosmotic flow is also changed. Consequently, a non-uniform flow field is developed along the channel (Figure 3). At ωt=π/2, the electric field is positive. Thus, the fluids near the walls move in the positive *x*-direction in the left half of the channel, since the zeta potential is negative in the left half. In contrast, due to the positive surface charge in the right half of the channel, the fluids near the walls move in the negative *x*-direction. These two opposite directional flows create two counter-rotating vortices in the upper part ($0\leq r/r_{0}\leq 1$) of the channel (Figure 3a). In fact, two counter-rotating vortices are formed at any angle θ because of the axisymmetric conditions. In other words, for a single period of surface potential, two oppositely rotating vortices (each one extending one half of the total length) are formed within the tube. So, if the tube is filled with two fluids in such a way that each fluid fills the entire cross-section and one-fourth length of the channel in an alternative patch, this design will have strong potential to enhance the mixing efficiency. Figure 3b shows the vector plot of the flow field at a nondimensional time, ωt=3π/4. This yields similar results to the previous case (Figure 3a) due to the similar conditions; however, the magnitude of the velocity components at the surface is reduced because of the reduced strength of the electric field. At ωt=π, the slip velocity at the wall reaches zero due to the no applied electric field (Figure 3c). However, bulk fluid motion is still observed at the center part of the tube due to the phase lag between fluids in the electric double layer and bulk fluids. The phase lag occurs due to the finite time requirement for momentum diffusion from the surface to the center line of the tube. This out-of-phase behavior is clearly visible at time ωt=5π/4 (Figure 3d), where velocity is negligible at the centerline of the channel but slip velocity at the channel surface is moderate. The centerline velocity approaches zero at ωt=1.26π and 2.26π. Based on these results, one can calculate the time lag as ~0.26π or 81.25 μs for an applied electric field frequency of 1.6 kHz.

When the electric field changes its direction, the velocity vector also switches its course (Figure 3d,e). For example, the distribution of velocity vectors at nondimensional time ωt=3π/2 appears to be opposite that at ωt=π/2 (Figure 3e vs. Figure 3a). It has been found that in EOF, the net flow and its direction depend on the overall strength of the zeta potential:

(19) $$\bar{\zeta }=\frac{\sum_{i}\zeta_{i}\Delta L_{i}}{\sum_{i}\Delta L_{i}}$$

where $\zeta_{i}$ and $\Delta L_{i}$ are the zeta potential and channel length at the i-th section, respectively [24]. Since the considered sinusoidal zeta potential has an overall strength of zero, the net flow in the current scenario is zero at any time.

### 3.2. Effect of Nondimensional Frequency

Previous works have shown that both electric field frequency and channel height have a similar effect on electroosmotic vortices [3]. Thus, it is wise to combine these two parameters to obtain a nondimensional frequency as $\Omega =\omega r_{0}^{2}/\vartheta$. This nondimensional frequency represents the ratio of the diffusion time scale, $t_{diff}=r_{0}^{2}/\vartheta$, to the period of the external electric field, $t_{p}=1/\omega$ [34]. The nondimensional frequency increases if either applied electric field frequency increases or the size of the tube increases.

**Figure 4:**
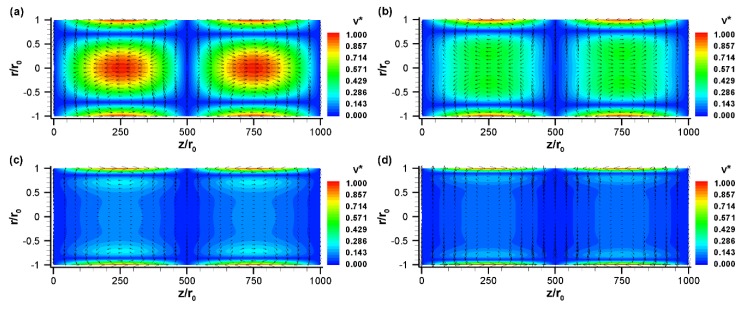
The normalized velocity,${\vec{v}}^{*}=\vec{V}/u_{HS}$ (vector plot) and its magnitude, $v*=\left|\vec{V}\right|/u_{HS}$ (contour plot) with a sinusoidal zeta potential distribution for various nondimensional frequencies: (**a**) Ω=2.5 (f=160 Hz), (**b**) Ω=25 (f=1.6 kHz), (**c**) Ω=125 (f=8 kHz), and (**d**) Ω=250 (f=16 kHz) at ωt=π/2. All other conditions are the same as those in Figure 3.

The effect of nondimensional frequencies is shown in Figure 4 for the sinusoidal zeta potential distribution case presented in Figure 3. Velocity vectors are presented for four different nondimensional frequencies: (a) Ω=2.5, (b) Ω=25, (c) Ω=125, and (d) Ω=250 at ωt=π/2. For a 100 μm diameter tube, these normalized frequencies correspond to f=0.16, 1.6, 8 and 16 kHz. As the nondimensional frequency increases, the pattern of the flow field changes significantly (Figure 4). At a low value of Ω (e.g., 2.5), the diffusion time scale is on the same order of magnitude of the external electric field period. As a result, the flow has enough time to propagate from the surface to the center of the channel, resulting in large vortices which extend from the surface to the center region of the tube (Figure 4a). However, as Ω increases from 2.5 to 25, these vortices are slightly shifted toward the surface and the bulk fluid velocity diminishes slightly (Figure 4b). When Ω increases from 25 to 125, the vortices are mainly confined to near the surface and the bulk fluid motion reduces significantly, as shown in Figure 4c. At a very high value of Ω (250 or higher), the bulk fluids are virtually motionless, despite the very fast oscillating flow occurring near the channel surface (Figure 4d). Thus, it is obvious from Figure 4 that the perturbed flow regime becomes smaller at higher nondimensional frequencies. The effect of nondimensional frequency on the flow field can be represented by damped viscous waves traveling away from the wall.

### 3.3. Effect of Surface Potential

**Figure 5:**
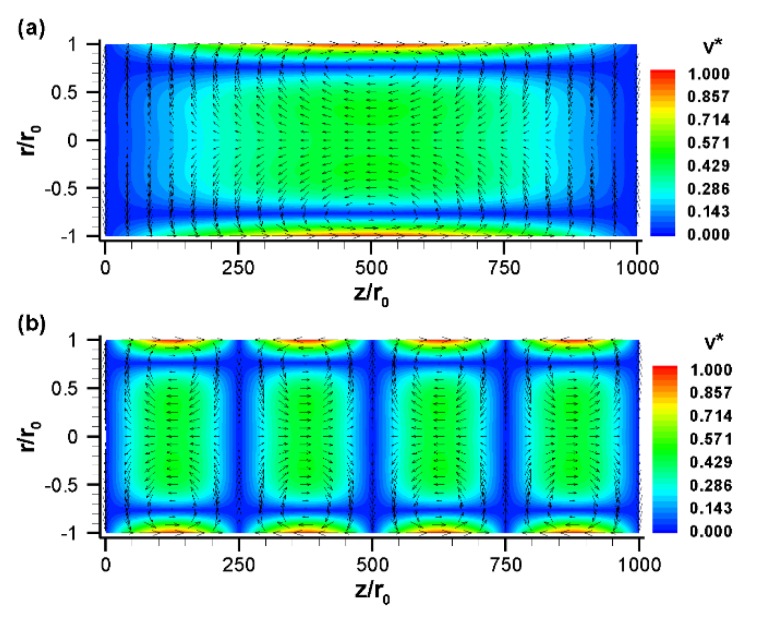
The normalized velocity, ${\vec{v}}^{*}=\vec{V}/u_{HS}$ (vector plot), and its magnitude, $v*=\left|\vec{V}\right|/u_{HS}$ (contour plot), with a sinusoidal zeta potential distribution with (**a**) k=π/L and (**b**) k=4π/L. Results are presented for ωt=π/2. All other conditions are the same as those in Figure 3.

Next, we studied the effect of surface potential distribution in the flow profile. As shown in Figure 5, the number of vortices formed within the channel can be controlled by the periodicity of the surface zeta potential distribution. With a half period (q=π/L), a large vortex is formed within the tube (Figure 5a), while with two periods (q=4π/L), four smaller vortices (each one extending one-fourth of the total length) are formed within the length of the tube (Figure 5b). Thus, a reduction in the surface potential periodicity reduces the number of vortices but extends the size of the vortices. Even though both scenarios (Figure 5a,b) provide an opportunity to enhance mixing through vortex formation, the higher periodicity case (Figure 5b) may be better for effective mixing because more vortices may yield a shorter mixing length. However, one has to be mindful about the sample loading to realize any positive outcome. For instance, in the case of half periodicity (Figure 5a), each mixing constituent needs to be loaded in each half of the channel. For example, fluid 1 should be loaded at length 0≤z≤L/2 and fluid 2 should be loaded at length L/2≤z≤L. In contrast, in the case of double periodicity (Figure 5b), each mixing constituent needs to be loaded at one-eighth of the channel length in an alternative patch. For example, fluid 1 should be loaded at 0≤z≤L/8, L/4≤z≤3L/8, L/2≤z≤5L/8, and 3L/4≤z≤7L/8, whereas fluid 2 should be loaded at L/8≤z≤L/4, 3L/8≤z≤L/2, 5L/8≤z≤3L/4, and 7L/8≤z≤L for effective mixing. Thus, our analytical results show that one can precisely control the flow field to achieve the desired level of mixing by modifying the zeta potential patterning on the tube surface.

### 3.4. Pressure Distribution

Although no external pressure was imposed for our proposed micromixer, pressure can be induced inside the channel to ensure the constant flow rate between different regions with different zeta potentials on the wall. This induced pressure can be quantified from the velocity profile. An analytical expression for pressure can be obtained from the modified Navier–Stokes equations. For instance, in the absence of an advection term, one can replace the axial velocity, i.e., the *z*-directional velocity component (Equation (16b)), in the *z*-component of the Navier–Stokes equation (Equation (6b)) to obtain the *z*-directional pressure gradient:

(20) $$\frac{\partial P}{\partial z}=\sum_{n=-\infty }^{\infty }A_{1,n}\left[i\rho \omega k_{n}I_{0}\left(k_{n}r\right)+\left(\mu l_{n}^{3}-\mu l_{n}k_{n}^{2}-i\rho \omega l_{n}\right)\frac{I_{1}\left(k_{n}r_{0}\right)}{I_{1}\left(l_{n}r_{0}\right)}I_{0}\left(l_{n}r\right)\right]\;e^{ik_{n}z}e^{i\omega t}.$$

The pressure distribution along the tube can be obtained by integrating with respect to z as follows:

(21) $$P=\sum_{n=-\infty }^{\infty }A_{1,n}\left[i\rho \omega k_{n}I_{0}\left(k_{n}r\right)+\left(\mu l_{n}^{3}-\mu l_{n}k_{n}^{2}-i\rho \omega l_{n}\right)\frac{I_{1}\left(k_{n}r_{0}\right)}{I_{1}\left(l_{n}r_{0}\right)}I_{0}\left(l_{n}r\right)\right]\;\frac{1}{ik_{n}}e^{ik_{n}z}e^{i\omega t}+P_{1}(r,t)$$

where $P_{1}$ is an integration factor, which can be a fixed value or a function of r and t. Now, by manipulating Equations (6a), (16a), and (21), we obtain:

(22) $$\frac{\partial P_{1}}{\partial r}=\sum_{n=-\infty }^{\infty }A_{1,n}\left[\left(-\rho \omega k_{n}+i\mu \frac{l_{n}^{4}}{k_{n}}-2i\mu k_{n}l_{n}^{2}+\rho \omega \frac{l_{n}^{2}}{k_{n}}+i\mu k_{n}^{3}\right)\frac{I_{1}\left(k_{n}r_{0}\right)}{I_{1}\left(l_{n}r_{0}\right)}I_{1}\left(l_{n}r\right)\right]\;e^{ik_{n}z}e^{i\omega t}.$$

The aforementioned equation indicates that $\partial P_{1}/\partial r$ might be a function of z,  r, and t. However, following numerical evaluation with a sinusoidal surface charge distribution (Equation (17)) and applied electric field, $\vec{E}=E_{0}sin\left(\omega t\right)=Im\left(E_{0}e^{i\omega t}\right)$, it is found that $\partial P_{1}/\partial r$ is zero in space and time (data not shown). Thus, the integration of Equation (22) will yield $P_{1}$ equal to a constant. Since we are not seeking the absolute pressure, we can neglect that constant, and quantify the gauge pressure distribution from Equation (21):

(23) $$P(r,t)=\sum_{n=-\infty }^{\infty }A_{1,n}\left[i\rho \omega k_{n}I_{0}\left(k_{n}r\right)+\left(\mu l_{n}^{3}-\mu l_{n}k_{n}^{2}-i\rho \omega l_{n}\right)\frac{I_{1}\left(k_{n}r_{0}\right)}{I_{1}\left(l_{n}r_{0}\right)}I_{0}\left(l_{n}r\right)\right]\;\frac{1}{ik_{n}}e^{ik_{n}z}e^{i\omega t}.$$

**Figure 6:**
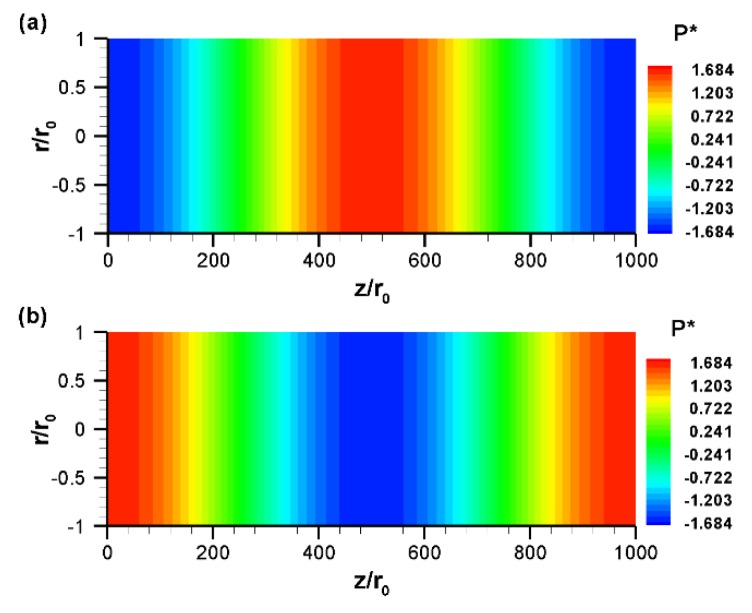
Distribution of normalized gauge pressure, $P*=Pr_{0}/\mu u_{HS}$, at different nondimensional times: (**a**) ωt=π/2 and (**b**) ωt=3π/2. All other conditions are the same as those in Figure 3.

The distribution of pressure at various nondimensional times is shown in Figure 6. From this figure, it can be seen that the pressure varies mainly in the axial direction. The reason behind this is discussed shortly. Since the flow field varies with time, the induced pressure also varies accordingly. For any particular time, for instance, ωt=π/2 (Figure 6a), an adverse (positive) pressure gradient is observed in the left half of the channel, while a favorable (negative) pressure gradient is formed in the right half of the channel. This induced positive pressure gradient tends to suppress electroosmotic flow, resulting in a mixed electroosmotic and pressure-driven flow, as shown in Figure 3a. This kind of velocity profile has also been obtained in an experimental study [35]. As the electric field direction switches, the flow field changes and, as a consequence, the pressure distribution is also switched, as shown in Figure 6b.

**Figure 7:**
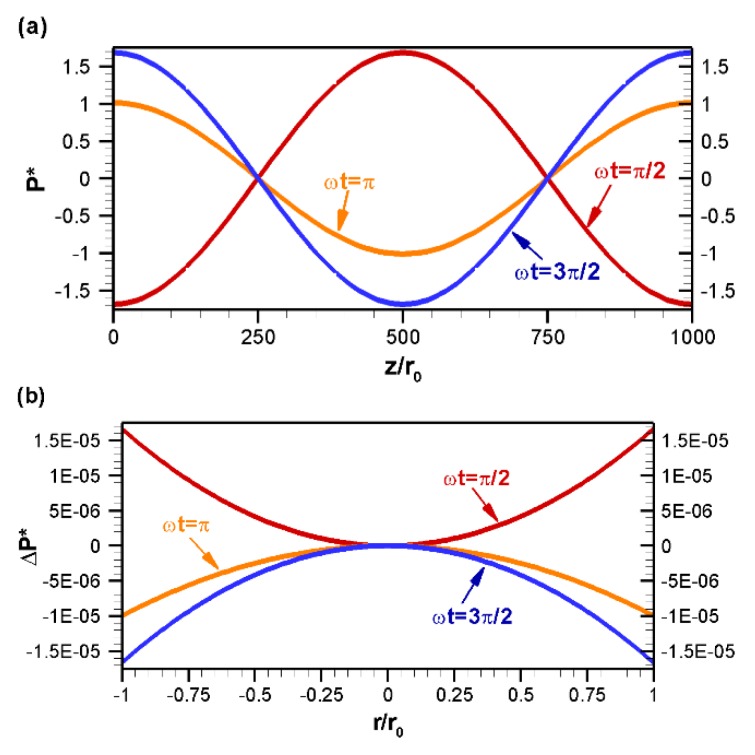
Distribution of gauge pressure, $P*=Pr_{0}/\mu u_{HS}$, at various nondimensional times: (**a**) Variation along the axial direction at $r/r_{0}=0$ and (**b**) along the radial direction at $z/r_{0}=500$, where $\Delta P*=P*-P*(r/r_{0}=0)$. All other conditions are same as those in Figure 3.

The axial variation of pressure is shown in Figure 7a for various nondimensional times, where pressure is obtained at the centerline of the tube. As seen from Figure 7a, for a sinusoidal variation of the zeta potential, the axial variation of pressure is also sinusoidal. However, there is a phase lag of 90° between the pressure distribution and zeta potential distribution. Thus, for a sine distribution of the zeta potential, the axial variation of induced pressure is cosine. At ωt=π/2, an adverse pressure gradient is observed in the left half of the channel, whereas a favorable pressure gradient is observed in the right half of the channel. As the electric field changes, the pattern of pressure variation in the axial direction also changes. For example, in contrast to ωt=π/2, at ωt=3π/2 a favorable pressure gradient is observed in the left half of the channel while an adverse pressure gradient is observed in the right half of the channel. It should be noted that in this work, a pressure gradient was also observed in the radial direction, however, the variation was very small in comparison to the axial variation and, hence, it was not captured in the pressure contour plot (Figure 6). The radial directional variation of pressure is shown in Figure 7b by line plot for various nondimensional times, where pressure is obtained along the radial direction of the channel at the axial location $z/r_{0}=500$ (z=L/2). Since the radial variation of pressure is very small, instead of gauge pressure, its change from the center point ($r/r_{0}=0,\;z/r_{0}=500$) is plotted in Figure 7b. This figure shows that the radial variation of pressure is several orders magnitude lower than that of the axial variation due to the low radial velocity. As the electric field changes direction, the pattern of pressure variation in the radial direction is also changed. For example, at ωt=π/2, the pressure variation in the radial direction follows a concave parabolic curve; whereas at ωt=3π/2 the pressure variation in the radial direction becomes a convex parabolic curve (Figure 7b).

## 4. Conclusions

Motivated by the growing interest in electroosmosis as a reliable non-mechanical strategy to control fluid motion and mix species in microfluidic devices, we analytically studied the time-periodic electroosmotic flow in cylindrical microchannels with a heterogeneous surface charge distribution. The analytical solution was derived by solving a simplified Navier–Stokes equation with Helmholtz–Smoluchowski slip boundary conditions at the wall. Although the derived analytical solution is valid for any periodic surface charge distribution, only the sinusoidal surface charge case was analyzed in this paper. The induced pressure field was also obtained from the velocity profiles. The results show that several vortices are formed inside the microchannel with a sinusoidal surface charge. These vortices change their pattern and direction as the external electric field changes with time. In addition, the dominance of the vorticity depends on the frequency of the external electric field and the size of the channel. As the electric field frequency or channel diameter increases, vortices are shifted towards the channel surface and the perturbed flow regions become smaller. Unlike the homogenous surface charge case, velocity variation in the radial direction was also observed in this paper. The results also show that the number of vorticities can be changed easily by changing the periodicity of the surface charge. Thus, the flow field can be precisely controlled with a specific surface pattern, which will provide the desired mixing efficiency.
